# Emotion computing using Word Mover’s Distance features based on Ren_CECps

**DOI:** 10.1371/journal.pone.0194136

**Published:** 2018-04-06

**Authors:** Fuji Ren, Ning Liu

**Affiliations:** Faculty of Engineering, Tokushima University, Tokushima, Japan; Nanyang Technological University, SINGAPORE

## Abstract

In this paper, we propose an emotion separated method(SeTF·IDF) to assign the emotion labels of sentences with different values, which has a better visual effect compared with the values represented by TF·IDF in the visualization of a multi-label Chinese emotional corpus Ren_CECps. Inspired by the enormous improvement of the visualization map propelled by the changed distances among the sentences, we being the first group utilizes the Word Mover’s Distance(WMD) algorithm as a way of feature representation in Chinese text emotion classification. Our experiments show that both in 80% for training, 20% for testing and 50% for training, 50% for testing experiments of Ren_CECps, WMD features get the best f1 scores and have a greater increase compared with the same dimension feature vectors obtained by dimension reduction TF·IDF method. Compared experiments in English corpus also show the efficiency of WMD features in the cross-language field.

## Introduction

Since the pace of modern life becomes faster and faster, people always work and live with high stress. From the report published by WHO, one in four people in the world will be affected by mental or neurological disorders at some point in their lives [1]. Thus, it’s momentous to make emotion computable for psychotherapy, health prediction or any other fields.

Emotions play an important role in successful and effective human-human communication [2]. There is also significant evidence that rational learning in humans depends on emotions [3]. With Google AI computer program AlphaGo beat Jie Ke at a three-game match in the 2017 Future of Go Summit, artificial intelligence drew the attention of the globe once again and will continue standing on top of the tides. This makes us recall the famous words noted by Marvin Minsky about the future of emotion computing: the question is not whether intelligent machines can have any emotions, but whether machines can be intelligent without any emotions [4].

In this paper, we propose an emotion separated method(SeTF·IDF) to assign the emotion labels of sentences with different values, which has a better visual effect compared with the values represented by TF·IDF in the visualization of a multi-label Chinese emotional corpus Ren_CECps. The separated method shows excellent ability of distinguishing sentences from multi-emotion labels, which can move data points away and avoid overlapping from each other. The moved multi-emotion points inspire us to be the first group to utilize the Word Mover’s Distance(WMD) algorithm as a way of feature representation in Chinese text emotion classification. Our experiments based on sentence level of Ren_CECps show that both in 80% for training, 20% for testing and 50% for training, 50% for testing experiments of Ren_CECps, WMD features get the best F1-scores of 0.318 and 0.31, where the baseline of TF·IDF are 0.196 and 0.204 respectively and the enhanced baseline of SeTF·IDF are 0.293 and 0.283. Compared with the same dimension feature vectors obtained by dimension reduction TF·IDF method, the WMD features get 3 times’ improvement based on F1-scores of 0.115 and 0.116. To speed up the calculation of WMD, we make changes of WMD algorithm, which gives a 16000 times decrease of time-consuming. For a better comparison, some experiments based on 20 newsgroup data set are also conducted. English corpus based experiments give an almost ten times promotion of F1-scores between WMD features(0.646) and dimension reduction TF·IDF method(0.076). Those above shows the efficiency of WMD features in classification of cross-language data sets and the WMD features have a strong ability in multi-emotion classification.

The remainder of this paper is organized as follows: Section 2 presents some related works. Section 3 gives the description of SeTF·IDF and describes the visualization of Chinese emotional corpus Ren_CECps. Section 4 goes for a comprehensive explanation of Word Mover’s Distance and the feature representation method. Section 5 illustrates the experimental configurations of two language data sets and draws the results in tables and graphs. In section 6, some discussions will be given. Section 7 presents the conclusions and future works.

## Related works

In 1997, “Affective Computing” was provided by Picard [3], which is of great importance and thereafter launched a new era in human emotion recognition and opinion mining. Accompanying with the blossoming of the Word Wide Web, it’s much easier to obtain text data to train a classifier. To show the abundant features of data, some interactive visualization methods were presented, like the most used parallel coordinates [5] and scatter-plot matrix [6] in attribute-decided data visualization. For the uncertainty of data labels, the measurement can be got in term of probabilities [7], which is useful in unTangle Map [8] for multi-label data visualization. As machine learning algorithms were introduced into NLP, a lot of annotated corpus without specific attribute values can be visualized by dimension scaling [9, 10], SVD [11], t-SNE [12]. With better visualization, the classification models can also be enhanced by integrating visual features and text features [13–15]. When it comes in large graph visualization, avoiding notes overlapping is another hot research topic. The principal method to solve this situation is elongating the distance within points, like force transfer [16] or changing the distribution of categories [17]. This is exactly what we do in this paper.

For similarity computing using a metric between two distributions, the Earth Mover’s Distance(EMD) [18] is one of the well-studied algorithms. By calculating the minimum cost that transform the distributions of color and texture into the other, the EMD can get better results for content-based image retrieval [19] and even can detect phishing web pages by visual similarity [20].

The most commonly used algorithms to represent the documents for similarity computing are statistic based algorithms like TF·IDF [21], LDA [22], or trained vectors using deep neural networks [23, 24]. In paper [25], Wan applied EMD into document similarity measurement successfully by decomposing the documents into a set of subtopics and using EMD to evaluate the similarity of many-to-many matching between the subtopics.

Limited by the NLP and machine learning algorithms, the pioneering studies in emotion computing were based on lexicons [26–28]. After years’ development, several annotated multi-emotion corpus were published [29–31]. Based on the emotion annotated corpus, the derived lexicon with multi-emotion tags can get higher F1 scores compared with traditional lexicon-based feature [32]. Relying on those emotional corpus, sentiment analysis can have a sub-field of emotion computing. A lot of machine learning algorithms were explored. SVM, Naive Bayes and Maximum Entropy are some of the most common algorithms used [33–35]. Some research using HMMs had also achieved better results [36, 37].

Emotion computing in Chinese has attracted many researchers due to the development of microblogging and tweet. Some studies in sentiment analysis of Chinese documents [38] turn to emoticon-based sentiment analysis [39]. But the studies of hidden sentiment association in contents [40, 41] are still one of the key points, like Chinese idiom emotion recognition [42], and can be especially important for measuring the mental healthy of humanity [43, 44]. To improve the study of affective computing in social networks, some standard corpus based on weibo data had been published [45–47]. Inspired by the excellent performance of deep neural network in image recognition, a lot of researches based on RNN [48], LSTM [49], CNN [50] for sentiment analysis had been done, works based on sentiment embeddings also get excellent results [51] and will attract more and more attention.

For estimating emotion of words that not registered in the lexicon, EMD can be applied to vectors of words and get higher accuracy compared with using only word importance value [52]. As the high time-consuming in EMD, the EMD based methods always limited into keywords or topics, not the full words. With the fast specialized solvers of EMD [53] was published, words fully transformed experiments can be carried out [54]. Those achievements facilitate the experiments in this paper.

## Visualization of Ren_CECps

For a multi-class corpus, we can make a 2D or 3D scatter-plot to have a good view of the distribution of the data. But for multi-class data with multi-label, to do the visualization with the same different colored points will make the 2D or 3D graph unreadable. Thus, how to match the multi-label information into a 2D or 3D graph is the key target need to be covered. In this paper, for visualizing the multi-label emotional corpus Ren_CECps, we propose an emotion separated TF·IDF method(SeTF·IDF) to represent each emotional category independently with different values. And to make a better 2D visualization, we use one of the stat-of-art dimension reduction algorithm t-SNE [12] to measure the low dimension distribution of Ren_CECps.

### Ren_CECps

Ren_CECps(can be accessed at http://a1-www.is.tokushima-u.ac.jp/member/ren/Ren-CECps1.0/DocumentforRen-CECps1.0.html) is an annotated emotional Chinese corpus using Chinese blog texts. The corpus was annotated in three levels: document level, paragraph level, sentence level [30]. Each level is annotated with its eight emotional categories(joy, hate, love, sorrow, anxiety, surprise, anger, expect) and corresponding discrete emotional intensity value from 0.0 to 1.0.

In this paper, the sentences without emotional labels are regarded as ‘neutral’ category. Table 1 shows the number of the sentences with different labels, the ‘neutral’ sentences were calculated in category one.

**Table 1:** The number of multi-label sentences in Ren_CECps.

label No.	total	one	two	three	four	five	six
sentence No.	36525	22751	11731	1847	175	15	6
per. (%)	100	62.2888	32.1177	5.0568	0.4791	0.0004	0.0001

### t-SNE

t-SNE(t-Distributed Stochastic Neighbor Embedding) is a technique for dimension reduction that is particularly well suited for the visualization of high-dimension datasets [12]provided by L.J.P van der Maaten and G.E Hinton. Compared with PCA algorithms, t-SNE computing the distributions of every nodes in the datasets and rebuilding the distribution of those nodes in two or three dimension space. To get the best approximate results, t-SNE uses KL divergence to measure the distance of the two distributions. In this paper, the t-SNE tool uses TSNE in sklearn [55] and the programm is followed a guidance blog written by Alexander Fabisch at http://nbviewer.jupyter.org/urls/gist.githubusercontent.com/AlexanderFabisch/1a0c648de22eff4a2a3e/raw/59d5bc5ed8f8bfd9ff1f7faa749d1b095aa97d5a/t-SNE.ipynb.

### Emotion separated representation

As mentioned above, considering the ‘neutral’ label as one emotion category, the total number of emotional categories needed to be calculated is nine. The keyword *word*_*i*_ of every sentences represented by TF·IDF can be calculated through Formula (1).

$$\begin{array}{c} tfidf=\frac{tf_{i}}{\sum tf_{i}}\times \log\frac{N}{df_{i}+1},i\in N \end{array}$$ (1)

In which, *tfidf* means the TF·IDF result of *word*_*i*_. *tf*_*i*_ means the term frequency of the calculated word. ∑*tf*_*i*_ means the frequency of the total words. *N* means the total sentences number. *df*_*i*_ means the total number of sentences which contain *word*_*i*_. From Formula (1), the conclusion we can get is that no matter what the words in the sentence are, the feature vector calculated though formula (1) for every annotated emotion labels of one sentence will be the same without any distinguishing.

The good news is that in Ren_CECps the emotion keywords of a sentence are annotated. Thus, for an annotated emotion keyword, we calculate its *tfidf* if and only if the emotion keyword has the given emotion category for a specific emotion label. In this way, we can generate a distinctive feature vector for each emotion label of a sentence. This method is named emotion separated TF·IDF(SeTF·IDF) method, and SeTF·IDF can be described as the formulas below:

$$\begin{array}{c} tfidf_{e_{j}}=\frac{S_{e}tf_{i}}{\sum tf_{i}}\times \log\frac{N}{df_{e}+1},i\in N,j\in \left[0,8\right] \end{array}$$ (2)

where, *e*_*j*_ ∈ [*joy*, *hate*, *love*, *sorrow*, *anxiety*, *surprise*, *anger*, *expect*, *neutral*]. In which, *tfidf*_*e*_*j*__ means the TF·IDF result of emotion keyword *word*_*i*_ in emotion category *e*_*j*_. *S*_*e*_ *tf*_*i*_ means the term frequency of emotion keyword *word*_*i*_ in emotion category *e*_*j*_. *df*_*e*_ means the total number of sentences which contain emotion keyword *word*_*i*_.

### The result of visualization

Following the algorithm below, the words without emotional labels annotated are calculated through formula (1), for the words with emotional label annotated, those are calculated by formula (2). The steps for visualization are as follows:

Using 2D data *M*_2_ and corresponding label list *l*, we can draw the 2D graph of Ren_CECps. The colors needed for every labels are listed as the sets:

Label: (*Anger*, *Anxiety*, *Expect*, *Hate*, *Joy*, *Love*, *neutral*, *Sorrow*, *Surprise*);

Color: (*red*, *blue*, *yellow*, *green*, *black*, *gray*, *orange*, *purple*, *pink*)

**Algorithm 1** The Procedure of Visualization

**Require:** *S*—sentences in Ren_CECps, *L*—labels of sentences

**Ensure:** 2D graph data

1: **function** Two-dimensionalization (*S*, *L*)

2:  **for** *sentence* ∈ *S* **do**

3:   calculate vectors by formulas (1) and (2);

4:   add vector into matrix *M* and corresponding *label* ∈ *L* into list *l*

5:  **end for**

6:  $M\overset{SVD}{\to }M_{50}$    ▹*M*_50_—50 dimensions’ row matrix reduced from *M*

7:  $M_{50}\overset{t-SNE}{\to }M_{2}$     ▹*M*_2_—2 dimensions’ row matrix reduced from *M*_50_

8:  **return** *M*_2_, *l*

9: **end function**

**Fig 1:**
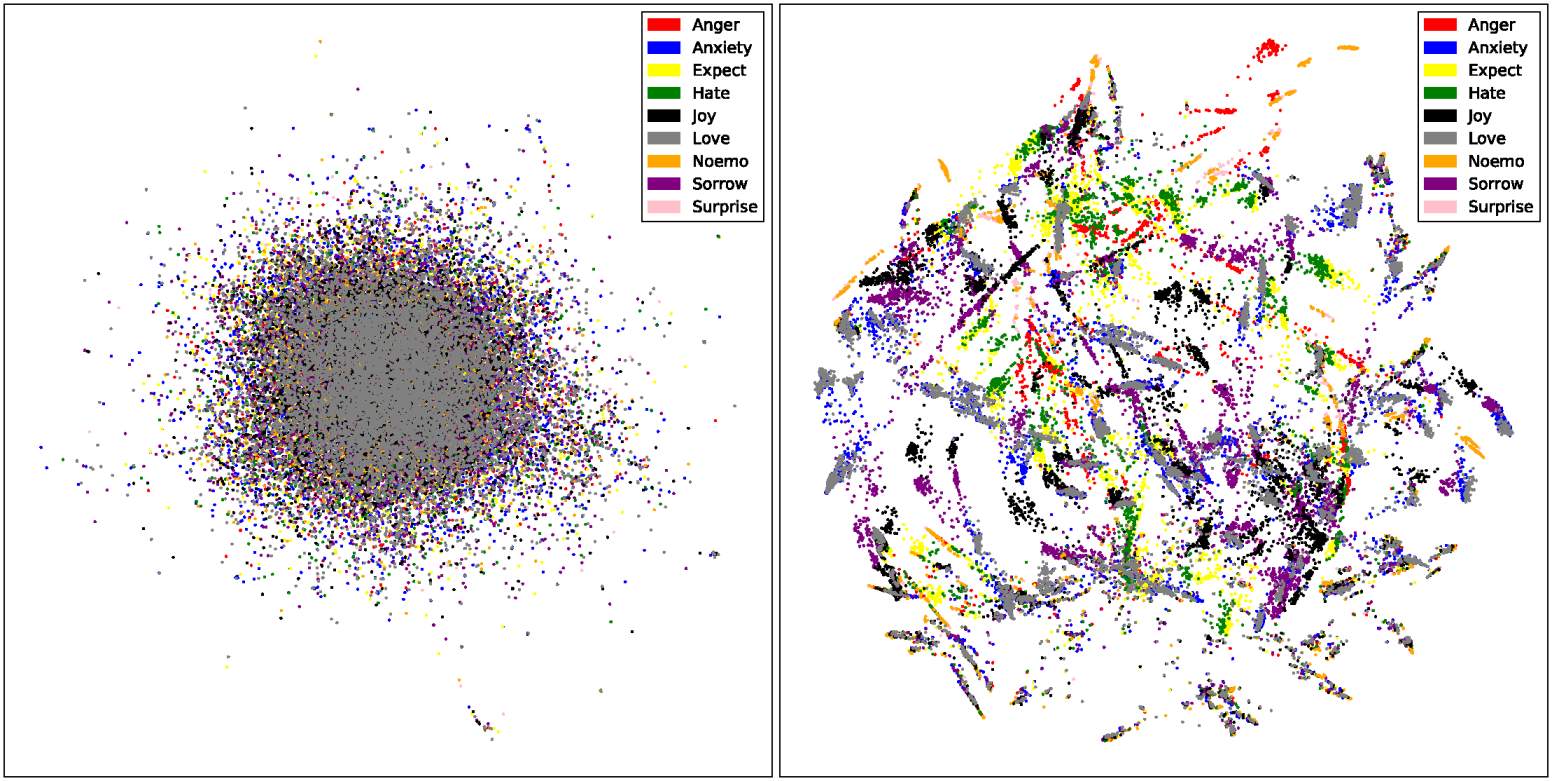
Visualization of Ren_CECps in traditional TF⋅IDF(left) and SeTF·IDF(right).

The Fig 1 shows the visualization of Ren_CECps in TF⋅IDF(left) and SeTF·IDF(right). We can find that the overlapping points in TF⋅IDF have been separated in SeTF·IDF. There are also some conclusions we can get:

- The elongated distances between every category of sentences and the distributions changed in SeTF·IDF indeed have a better visual result compared with TF·IDF.
- “Love” points have the similar distribution compared with “Anxiety” points;
- Most of the “Sorrow” points come with no other emotional points embedding into their group.
- Sentences may have completely opposite emotion categories. In some clusters, there are pairs like “Sorrow and Joy”, “Hate and Love”.
- Fuzziness emotion categories such as “Expect” have the most frequency to appear with other emotion categories.

Based on those features, we can have a more clear vision of Ren_CECps. For a better view, we also make a 3D visualization result for the emotional corpus at http://a1-www.is.tokushima-u.ac.jp/data_all/.

## Word mover’s distance feature representation

The visualization graphs in Fig 1 show the remarkable progress derived from the changed distances among emotional points. It seems like a lot of words walk away from each other and which makes the sentences heavy fog in the left being blown over into air circulation. This inspires us if we can let the words “walking” in the algorithm, maybe we will get better results in classification. Following the desire, we focused on transportation problem in NLP, and found word mover’s distance algorithm.

**Word Mover’s Distance** The word mover’s distance(WMD) [54] is a good distance measure came from earth mover’s distance(EMD) [19]. The EMD problem can be solved as transport problem. As in WMD, the distance between two text documents A and B is the minimum cumulative distance that words from document A need to travel to match exactly the point cloud of document B [54]. The transportation matrix between documents A and B can be described as formula (3) below:

$$\begin{array}{c} \begin{array}{cc} & \begin{array}{ccccc} {\text{word}}_{1} & \cdots & {\text{word}}_{i} & \cdots & {\text{word}}_{n}\\ d_{1} & \cdots & d_{i} & \cdots & d_{n} \end{array}\\ \begin{array}{cc} {\text{word}}_{1}^{\prime } & d_{1}^{\prime }\\ \vdots & \vdots \\ {\text{word}}_{j}^{\prime } & d_{j}^{\prime }\\ \vdots & \vdots \\ {\text{word}}_{m}^{\prime } & d_{m}^{\prime } \end{array} & \left[\begin{array}{ccccc} \omega_{1,1} & \cdots & \omega_{1,i} & \cdots & \omega_{1,n}\\ \vdots & \ddots & \vdots & \ddots & \vdots \\ \omega_{j,1} & \cdots & \omega_{j,i} & \cdots & \omega_{j,n}\\ \vdots & \ddots & \vdots & \ddots & \vdots \\ \omega_{m,1} & \cdots & \omega_{m,i} & \cdots & \omega_{m,n} \end{array}\right] \end{array} \end{array}$$ (3)

Where, {**word**_*i*_} and {${\text{word}}_{j}^{\prime }$} represent the words in document A and document B respectively. {*d*_*i*_} and {$d_{j}^{\prime }$} mean the term frequencies of the corresponding words. *ω*_*j*,*i*_ is the distance between ${\text{word}}_{j}^{\prime }$ and **word**_*i*_, especially the distance is undirected.

To measure the distances of two words, every words are represented as vectors provided by trained *word*2*vec* embedding matrix **V**, and the distances can be calculated by Euclidean distance in formula (4):

$$\begin{array}{c} \omega_{j,i}=∥V_{j},V_{i}{∥}_{2},V_{j,i}\in V \end{array}$$ (4)

Let **T**_*ij*_ where *i* ∈ [1, *n*], *j* ∈ [1, *m*] be the number of **word**_*i*_ in document A which transports into ${\text{word}}_{j}^{\prime }$ of document B. In this way, $\sum_{j=1}^{m}\sum_{i=1}^{n}T_{ij}$ denotes the total numbers of words in document A transporting into the words of document B. On the contrary, $\sum_{i=1}^{n}\sum_{j=1}^{m}T_{ji}$ means the reverse direction of the transportation.

Thus, the WMD of documents distance measurement can be described as an optimization problem in formula (5), and the minimum result is the distance of two documents.

$$\begin{array}{cc} \min & \;\sum_{j=1}^{m}\sum_{i=1}^{n}T_{ij}\omega_{j,i}\;\;\\ \text{subject}\;\text{to:} & \;\sum_{j=1}^{m}\sum_{i=1}^{n}T_{ij}=\sum_{j=0}^{m}d_{j}^{\prime },\\ & \sum_{i=1}^{n}\sum_{j=1}^{m}T_{ji}=\sum_{i=0}^{n}d_{i},\\ & \;T_{ij}\geq 0. \end{array}$$ (5)

Here is an example of calculating the similarities of two target sentences S1, S3 with a standard sentence S2 in WMD and TF·IDF, the sentences are all tokenized and split with blank space:

    S1: “风和日丽.(English: Sunny Days.)”

    S2: “天气 很 好.(English: It’s a good day.)”

    S3: “今天 下 雨.(English: It’s raining today.)”

We use the *cosine* function to calculate the vectors represented in TF·IDF as the similarity measurement and the WMD are calculated following the “word mover’s distance in python” at http://vene.ro/blog/word-movers-distance-in-python.html published by vene&Matt Kusner [54]. Assuming *sim*() formula as the similarity between two sentences, we can describe the results below:

*WMD*: *sim*(*S*1, *S*2) = 0.75, *sim*(*S*3, *S*2) = 0.82.

*TF*⋅*IDF*: *sim*(*S*1, *S*2) = 1.0, *sim*(*S*3, *S*2) = 1.0.

In TF·IDF, S1 and S3 get the same similarity results. In WMD, S1 gets a lower result compared with S3, this means S3 is farther away from s2 than S1 from S2. Or can be said that S2 is more similar to S1 than S3, and this matches the ground truth.

As the example shows, WMD has an ability to measure the semantic difference between sentences. Thus we can use several selected sentences as a core dataset, the samples in the entire corpus can be represented by its similarities with all of the sentences in the core dataset. And we will verify this feature representation method in the next section.

## Experiments and results

We evaluate the WMD features in SVM [56] model on Ren_CECps, and regard TF·IDF [57], SeTF·IDF as baseline and enhanced baseline respectively. To be comprehensive, two low dimensional feature representation methods will be evaluated. For a better comparison, an English corpus based experiment is further added.

### Dataset and setup

**Ren_CECps** The corpus is divided into nine single label data sets. Sentences with multi-label will be replicated in every categories. We select 200 sentences from every nine emotion categories randomly as the seed corpus, naturally, the dimension of the WMD features is 1800. Based on the divided corpus, two ways of selecting subsets for the experiments will be executed: one is 50% of the data for training and the rest 50% of the data for testing; the other one is 80% of the data for training and the rest 20% of the data for testing, the selection is random.

**20 newsgroups data set** We utilized the split “train” and “test” data sets [58] provided by sklearn tools at http://scikit-learn.org/stable/datasets/twenty_newsgroups.html. All of the “headers”, “footers” and “quotes” in data sets are removed. The number of the seed documents for the 20 news categories is 100 and the selection is also random from “train” subset.

**Word embeddings** The word embeddings used in this paper are different with languages. For Chinese, we merged two additional Chinese data sets(sougouCA at https://www.sogou.com/labs/resource/ca.php: A Chinese news corpus published by Sougou Lab [59] and People’s Daily data set: We collected 11,355 days’ news data from 1980.01.01 to 2016.02.14 through the Internet) into Ren_CECps to train a 200 dimension word embedding using *gensim* [60] which is a free python library containing the approach in [23]. For English, a pre-trained embedding at https://code.google.com/archive/p/word2vec/ will be utilized for the experiments, which contains 300-dimension vectors for 3 million words and phrases. Both in Chinese and English experiments, the words not in the embeddings will be determined as zero vectors.

### Fast computing

In this paper, the main calculation process is following the program used in examples computing of section 4. The EMD has a best average time complexity of *O*(*N*^3^log *N*) [53], where *N* denotes the vocabulary length. This means the lower scale of words, the faster the computing will be. We continue to use **V** as the word embedding, the pseudo-code of calculating WMD of documents used in [54] can be show in Algorithm 2, a pseudo-code of fast WMD for comparing is presented in Algorithm 3 side by side.

As can be shown below, the matrix **TD** needed for WMD in algorithm 2 is exported from the whole documents of corpus, this is a fully vocabulary length matrix with dimension of tens of thousands in Chinese or hundreds of thousands in English. In algorithm 3, we export the matrix **TD**’ only from two documents which need to be calculated. This makes the dimension of **TD**’ far less than **TD**, and restricts the dimension into one hundred. For each computation of WMD, though the T-D matrix **TD**′ and distance matrix **M**′ are both needed to be recomputed within every loop process, the fast WMD still has a lower computational complexity compared with the exponential order complexity of EMD in step 8 of algorithm 2.

**Algorithm 2** WMD             **Algorithm 3** fast WMD

1. **for** corpus *D* **do**               1. **loop** *i*, *j* in index

2.  T-D matrix **TD** ← *D*            2.  **for** *d*_*i*_, *d*_*j*_ in corpus *D* **do**

3. **end for**                     3.   T-D matrix **TD**′ ← [*d*_*i*_, *d*_*j*_]

4. **for** words in **TD** **do**               4.  **end for**

5.  distance matrix **M** ← **TD**,**V**          5.  **for** words in **TD**′ **do**

6. **end for**                     6.   distance matrix **M**′ ← **TD**′,**V**

7. **loop** *d*_*i*_, *d*_*j*_ in corpus *D*              7.  **end for**

8.  **return** emd(**TD**[*i*],**TD**[*j*],**M**)             8.  **return** emd(**TD**′[0],**TD**′[1],**M**′)

9. **end loop**                  9. **end loop**

To verify the improvement, we make three groups of experiments. Each group is a ten times’ computing based on 10 pairs of sentences which are selected randomly from Ren_CECps. The comparison of time-consuming is illustrated in Table 2.

**Table 2:** The comparison of time-consuming in WMD and fast WMD.

Groups	Case 1	Case 2	Case 3
	per 10 times(s)		
**WMD**	632.545	646.700	646.237
**fast WMD**	0.047	0.042	0.031
rate	**16000 times**		

**Parallelization** Though the fast WMD algorithm has a 16000 times improvement of computation efficiency compared with WMD algorithm, it’s still too slow for our experiments, so we parallelize the fast WMD model using 10-12 processes in 8 servers. To be consist, the words of WMD written in the rest part of this paper are all in the meaning of the fast WMD algorithm without special explanation.

### Evaluation measures

In this paper, the evaluation is measured by F1-score:

$$\begin{array}{c} F1-score=\frac{2*precision*recall}{precision+recall} \end{array}$$ (6)

where:

$$\begin{array}{ccc} precision & = & \frac{tp}{tp+fp}\\ recall & = & \frac{tp}{tp+fn} \end{array}$$ (7)

In which, *tp* is the number of true positive, *fp* is the number of false positive and *fn* is the number of false negatives. In this paper, both of *precision* and *recall* are calculated in ‘macro’ model using *metrics* package in sklearn at http://scikit-learn.org/stable/modules/classes.html#module-sklearn.metrics.

### Results

The experiments are arranged based on 50321 sentences(split in categories) of Ren_CECps and 18846 documents of 20 newsgroup. We use a Linear Support Vector Machine library at http://scikit-learn.org/stable/modules/generated/sklearn.svm.LinearSVC.html for our classification experiments. All of the SVM programs are running upon the default configuration.

We will make some classification experiments among TF·IDF, SeTF·IDF, WMD and a sentence embedding method which is one of the stat-of-the-art methods trained by sent2vec [61]. To make a full comparison with the feature’s dimensions, some low dimensional feature representation based experiments with TF·IDF and selected seed sub-corpus of the two language corpus will be carried out. For a better comparison for whether word embedding has a more important influence above WMD or not, we add another experiment in which a method combined TF·IDF and word embedding will be experimented. Here are some abbreviations using all through this section:

- **1v1:** The experiments based on the 50% of Ren_CECps for training and the rest 50% of Ren_CECps for testing;
- **4v1:** The experiments based on the 80% of Ren_CECps for training and the rest 20% of Ren_CECps for testing;
- **20 news:** The experiments based on the training and testing data of 20 newsgroup data set;
- **TF·IDF_1800_:** A low dimensional feature representation method, using SVD at http://scikit-learn.org/stable/modules/generated/sklearn.decomposition.TruncatedSVD.html to reduce TF·IDF feature vectors into 1800 dimensions;
- **TF·IDF_2000_:** The 2000 dimensions feature representation method reduced from TF·IDF feature vectors;
- **Seed_TF·IDF:** Using cosine function to calculate the similarity between target data and seed sub-corpus, in which the final similarities will be the feature vectors of training and testing data. In these experiments, all of the data are initialized by TF·IDF. We assume (*v*_1_, ⋯, *v*_*i*_, ⋯, *v*_*n*_) as the TF·IDF represented sub-corpus, in which *v*_*i*_ is the TF·IDF vector. *t*_*j*_ means the TF·IDF vector of training and testing data. Thus, the final feature vectors of training and testing data can be calculated as this function: (cos(*v*_1_, *t*_*j*_), ⋯, cos(*v*_*i*_, *t*_*j*_), ⋯, cos(*v*_*n*_, *t*_*j*_))
- **TF·IDF_word2vec:** The enhanced TF·IDF method which uses the word embeddings trained by word2vec as the weights for the corresponding words. The feature vectors used in this experiment are exported by the multiplication of TF·IDF and the embedding matrix.
- **sent2vec:** In this experiment, we use sentences embeddings trained by sent2vec tool at https://github.com/epfml/sent2vec as feature vectors. Every documents of 20 newsgroup data set are converted into one line files. The output dimension of sentence embeddings is 700.

**Results pre-processing** As the results computed by WMD contain the “NaN” data. In order to fit data into SVM, we convert all of the “NaN” data into integer of zero. In discussion section, we will explain the reason.

**Fig 2:**
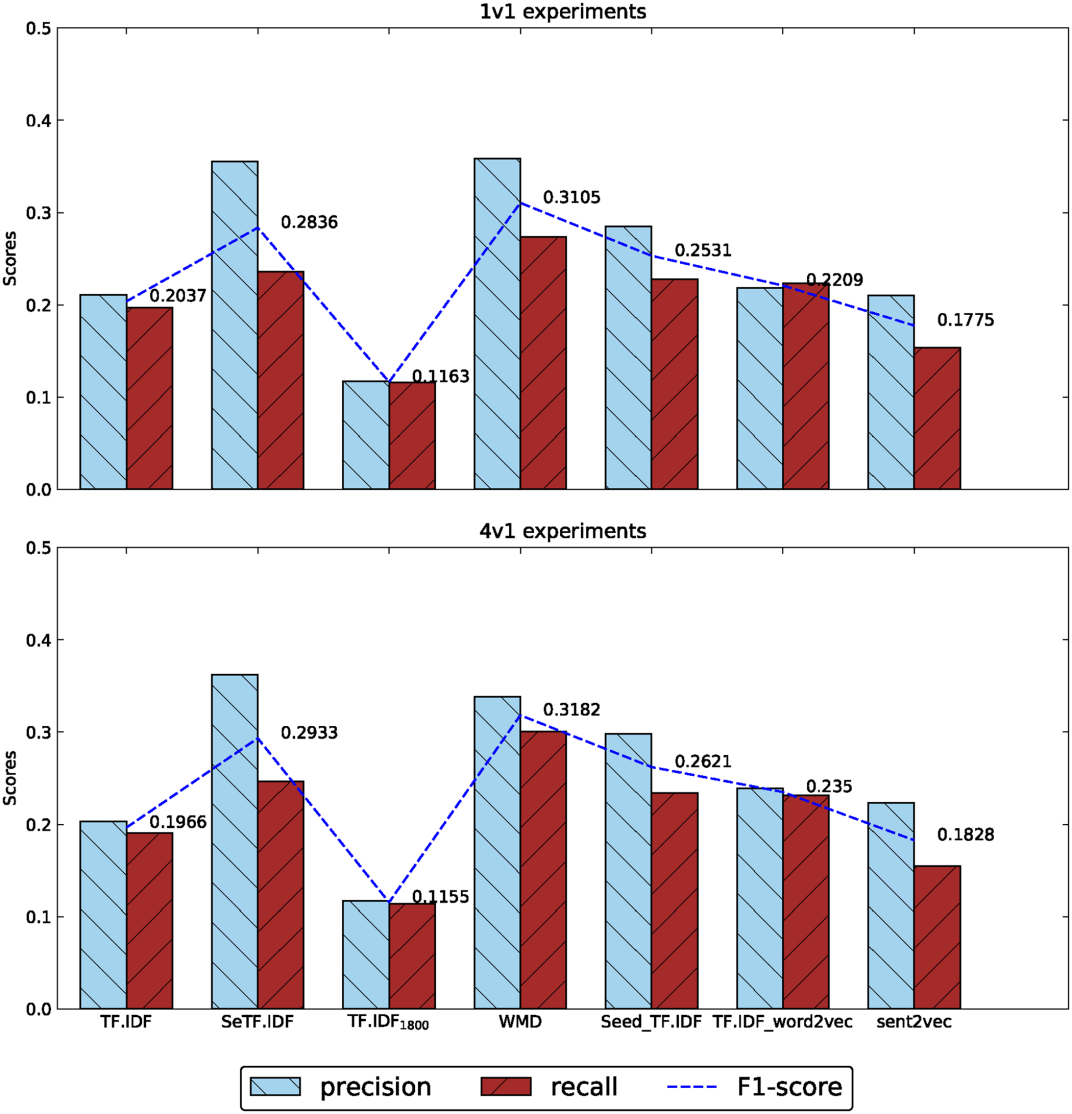
The results of 1v1 and 4v1 experiments.

**Fig 3:**
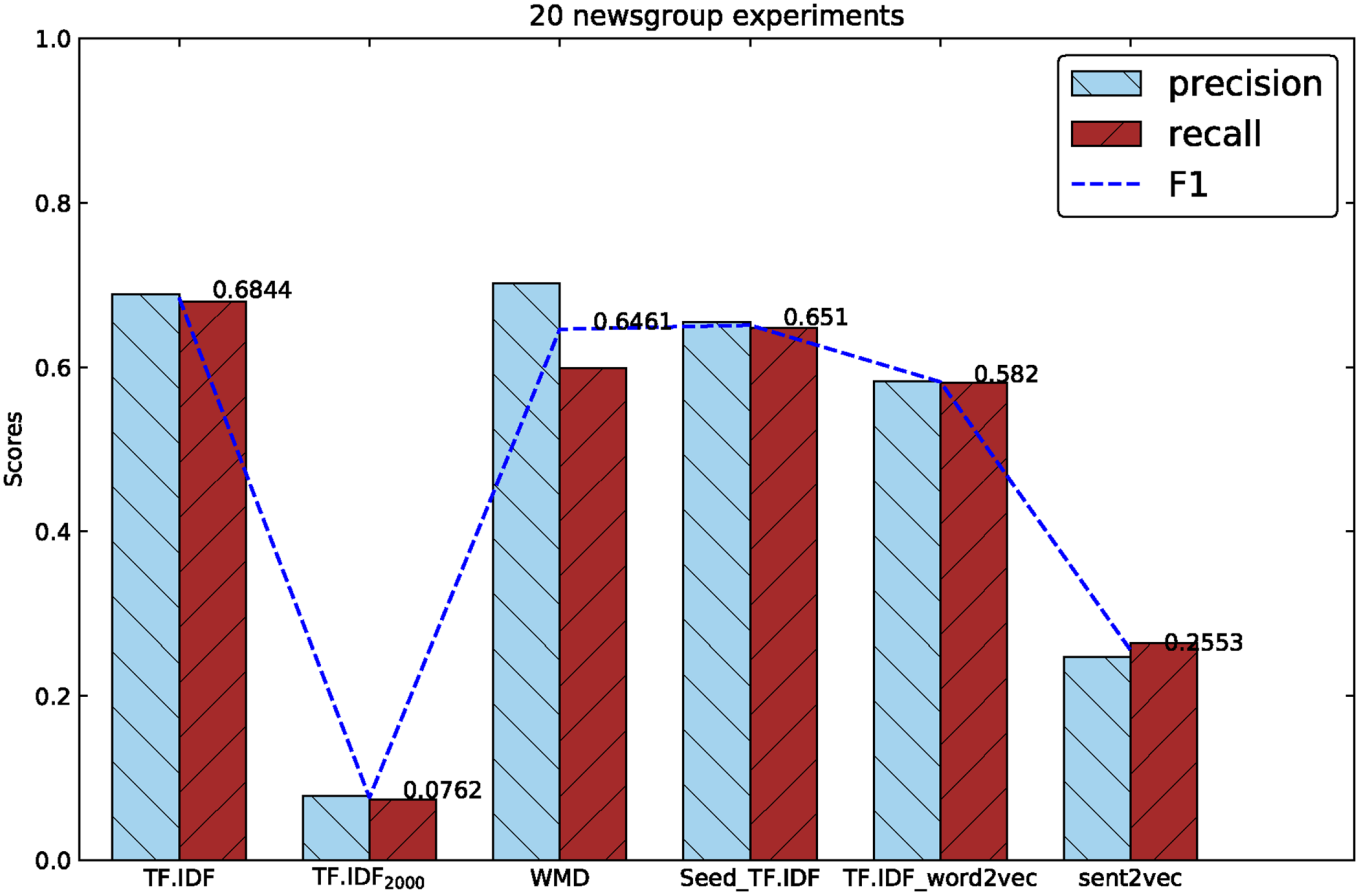
The results of five methods in 20 newsgroup experiments.

Table 3 shows the results of classification experiments based on the feature representation methods mentioned above, the best and worst results of three experiments are all marked in bold. According to the results, we drew some histogram graphs below. Fig 2 shows the comparison graph between 1v1 and 4v1 experiments, and Fig 3 shows the results in 20 newsgroup.

**Table 3:** The results of experiments on Ren_CECps and 20 newsgroup.

Type	Algorithm	Precision	Recall	F1-score
**1v1**	TF·IDF	0.210819957	0.197094468	0.203726295
	SeTF·IDF	0.355171204	0.236033564	0.283598272
	TF·IDF_1800_	0.116894026	0.115778614	**0.116333646**
	WMD	0.358587638	0.273787523	**0.310501826**
	Seed_TF·IDF	0.284783174	0.227757698	0.253098099
	TF·IDF_word2vec	0.218511086	0.223298753	0.220878979
	sent2vec	0.209975675	0.153755042	0.177520441
**4v1**	TF·IDF	0.203162567	0.190372868	0.196559888
	SeTF·IDF	0.361914601	0.246556037	0.293300035
	TF·IDF_1800_	0.117098454	0.113943072	**0.115499216**
	WMD	0.338477937	0.300256706	**0.318223762**
	Seed_TF·IDF	0.29824698	0.233749726	0.262088652
	TF·IDF_word2vec	0.238826227	0.231257587	0.234980977
	sent2vec	0.223046830	0.154791461	0.182754080
**20 news**	TF·IDF	0.688655922	0.680110185	**0.684356377**
	TF·IDF_2000_	0.078399628	0.07408649	**0.07618206**
	WMD	0.701975748	0.598521007	0.646133456
	Seed_TF·IDF	0.654635361	0.647471893	0.651033922
	TF·IDF_word2vec	0.582910280	0.581137014	0.582022296
	sent2vec	0.246903835	0.264207205	0.255262622

In Fig 2, we can find that both in 1v1 and 4v1 experiments, the WMD algorithm gets the best classification results, which even higher than the manual emotion separated method of SeTF·IDF over about three percentage points. When compared with the same dimensional features, the WMD shows strong information representation capability, and gets 20% higher score than the low dimension TF·IDF_1800_ and 5% higher than similarity representation method of Seed_TF·IDF.

But in Fig 3, the WMD method is knocked off in English news experiments. The 20 newsgroup based experiments get the best results in TF·IDF, and WMD gets nearly the same F1-score with Seed_TF·IDF. Both of the two methods are 5% lower than TF·IDF model. One thing makes us excited is WMD method is still higher than the low dimension TF·IDF_2000_ and gets almost 10 times promotion on F1-score.

We can find the TF·IDF_word2vec method gets the F1-score of 0.2209, 0.235 and 0.582 respectively in Figs 2 and 3, and are all lower than the results got by the WMD method of 0.3105, 0.3182 and 0.6461. The sent2vec experiments haven’t got the best results than other methods, the F1-scores are only better than TF·IDF_2000_ and TF·IDF_1800_.

In Figs 2 and 3, all of the low dimension feature representation methods reduced from TF·IDF model get the worst results. But selected seed corpus based similarity representation algorithm gets higher results in 1v1 and 4v1 experiments of Chinese corpus than TF·IDF model, gets lower results in 20 newsgroup experiments of English corpus in the contrary. After digging into the feature dimensions of those methods, we found the dimensions of TF·IDF vectors in Chinese and English corpus are 30,000 and 130,000 in integer respectively. The dimensions of WMD method in the two corpus are 1800 and 2000 separately as mentioned before. Computing the rate of dimension reduction of WMD, the Chinese corpus got a reduction rate of 17:1 and English corpus got a reduction rate of 67:1, this may explain why WMD perform better in Chinese corpus than in English corpus. The same situation can be found in the results between TF·IDF and dimension reduced TF·IDF of TF·IDF_1800_ and TF·IDF_2000_: In 1v1 and 4v1 experiments, the F1-scores of TF·IDF_1800_ drop two times compared with TF·IDF(0.22 to 0.11, 0.196 to 0.115), while in 20 newsgroup, F1-scores of TF·IDF_2000_ decline nine times compared with TF·IDF(0.68 to 0.07).

## Discussion

**Difficulty in SeTF·IDF** Though SeTF·IDF can match sentences into different emotion dimensions, the method is based on priori knowledges annotated in corpus. This means we cannot use SeTF·IDF to match a new sentence or document into multi-emotion dimensions due to lack of no emotional keywords annotated manually. That’s why we use SeTF·IDF as an enhanced baseline method. It’s an idealized results. The importance is this visualization algorithm makes us having a clearer visual results, and changes our way of thinking in training multi-label data.

**“NaN” conversion** Both of the results of the Chinese and English corpus computed by WMD contain “NaN” data. This makes the data disable to train in SVM model. Parsing the sentence pairs which “NaN” data happened, we found the “NaN” data always appear in short sentences or documents, and the target data are the same with the seed data, like “好! (English: good)” and “不知(English: I don’t know.)” in Chinese corpus, “Thanks!” and “It’s there…” in English corpus. One special situation is the documents with a lot of messy script codes in 20 newsgroup data set, and these messy codes will result in “NaN” data.

Having known the contents leaded to error, we make two ways to convert the “NaN” data. One is replacing the “NaN” data with “0”, and the other one is “1”. The reason for “0” conversion is that the pairs of sentences are the same, and in distance meanings of WMD, “0” is the most suitable and practical; But considering the similarity vector, “0” elements are useless, and may cause information loss, “1” conversion maybe better.

To verify which one is suitable, we convert the “NaN” data to “0” and “1” independently in both 1v1 and 4v1 experiments. The “1” conversion data gets 0.291 and 0.305 in F1-scores in 1v1 and 4v1 experiments respectively, a bit lower than “0” conversion of 0.310 and 0.318. Thus, we choose “0” conversion finally in all of the experiments.

**The opposite results in Chinese and English data sets** In 1v1 and 4v1 experiments, the WMD method gets the best results. On the contrary, in 20 news, the TF·IDF gets the best result, and Seed_TF·IDF gets the second, third is WMD. One reason is the reduction rates mentioned above of the two language corpus are different. The other reason maybe the word embeddings of English used in the experiments have more missing words than Chinese embeddings, this can explain why TF·IDF_word2vec gets higher results and indeed should be higher than TF·IDF in Chinese corpus, but gets fourth rank in English corpus and almost 10% lower than TF·IDF.

## Conclusion and future work

The experiments show some enlightening conclusions based on the cross-language corpus.

1. Distance changed by the emotion separated method can get higher visual performance in multi-label emotional corpus;
2. The WMD algorithm is indeed efficient for classification.
3. Different language has different information density of words. Thus can influence the results of feature representation methods.
4. In Chinese corpus, owing to the high information density of words, for a certain degree of feature representation reduction, it’s good for the classifier to training models and can help improve results; For English corpus, due to the lower information density of words, the same degree for reducing features may not good for model training and needs more experiments to find the best degree.

Though we had given a fast computing frame of the original WMD algorithm, the calculation process is still time-consuming, to generalize the model, a more fast computing improvement is needed, and this will be our future work. For the emotion classification experiments, the just over 30% F1-scores cannot supply the emotional recognition applications, we will continue to improve this field.
